# Benefits of serum protein electrophoresis as part of hematopoietic stem cell donor clearance

**DOI:** 10.1002/jha2.997

**Published:** 2024-08-29

**Authors:** Laura Kilinc, Burkhardt Schleipen, Karen Ende, Deborah Buk, Alexander H. Schmidt, Isabel Auer, Thilo Mengling

**Affiliations:** ^1^ DKMS Group Tubingen Germany; ^2^ DKMS Donor Center Tubingen Germany

**Keywords:** hematopoietic stem cells, MGUS, stem cell transplantation

To be cleared for hematopoietic stem cell (HSC) donation, potential donors must undergo a physical examination including blood testing to test their health status and eligibility to donate, ensuring safety for the donor and limiting the risk of transmitting infectious, genetic, or neoplastic diseases from the donated HSC product to the recipient. In Germany, the applicable national standards [1] require additional donor blood testing with serum protein electrophoresis (SPEP), to exclude the presence of monoclonal gammopathy of undetermined significance (MGUS) as part of this physical examination.

MGUS is a premalignant plasma cell disorder in which plasma cells produce incomplete or non‐functional monoclonal antibodies (paraproteins). Patients with MGUS have a life‐long risk of developing multiple myeloma (MM), smoldering myeloma (SMM), or a related malignant disorder. The condition is usually discovered by the presence of serum monoclonal protein (M protein) that forms a peak (M gradient), usually in the gamma‐globulin fraction, in SPEP [2]. MGUS is found in more than 3% of the population aged 50 years and older, its prevalence increases with age, and it is more often found in men than in women [3, 4]. The risk for MGUS patients to develop MM, SMM or a related malignant disorder is about 1% per consecutive year [4, 5].

Even if large quantities of plasma cells should not be transferred during HSC transplantation, a transfer of premalignant clonal cell populations to the recipient cannot be excluded. Transmission of MGUS has already been shown in solid organ transplantations [6]. In addition, blood‐borne malignancies have been transferred by HSC transplantation [7, 8]. Therefore, registered donors with MGUS are ineligible for HSC donation and need to be identified and excluded during the physical examination with SPEP prior to donor clearance for HSC donation. However, in many countries, donor testing for MGUS is not carried out prior to HSC donation. Our findings highlight the need to include MGUS testing with SPEP for potential HSC donors prior to HSC collection as a standard to ensure both donor and specifically patient safety.

In this work, we present data from DKMS Germany within a time period of 13 years (2009–2022) in which potential HSC donors were screened for MGUS during the physical examination prior to HSC collection. Based on these data, we analyzed the effect of MGUS testing with SPEP at this process step and discuss implications for HSC donation.

1

Since 2009 (observation period: January 2009 until December 2022), DKMS Germany has had all potential HSC donors who were requested for donation tested for MGUS as part of the physical examination, taking place within 30 days prior to the planned collection date at the collection center. The method used to detect MGUS was SPEP. Immunofixation electrophoresis (IFE) was used to further differentiate the paraproteins. The total serum protein content was also determined on a routine basis. Furthermore, abnormal SPEP curves can further reveal other diseases such as antibody deficiencies.

During the physical examination, no cases of MM or SMM were detected, but donors presenting abnormal SPEP curves (M gradient) indicating MGUS (defined as M protein concentration < 3 g/dL and absence of end‐organ damage characterized by CRAB criteria (hypercalcemia, renal insufficiency, anemia, bone lesions)) [4, 9] were not cleared to proceed for donation. Cases were retested for MGUS via their general practitioner to either confirm or refute the original diagnosis. All deferred cases between 2009 and 2017 were included in a follow‐up study and were routinely followed up for ten years (as are all actual stem cell donors) but the proportion of deferred donors which did not respond was high (around 40% of deferred cases and 57% of MGUS cases did not respond).

2

In the period from January 2009 to December 2022, 4344 of 97,938 requested donors (4.4%; 1511/29,003 female, 2833/68,935 male), were not cleared to proceed to HSC donation. Besides the main reasons such as poor vein status or an enlarged spleen, another common reason for donor deferral was the diagnosis of potential MGUS: In 143 potential donors (0.15%; 38 females and 105 males) abnormal paraprotein was detected, corresponding to 3.3% of all non‐clearances. In three cases (one MM and two SMM) disease progression was confirmed diagnostically within 1 year. Regardless of donor sex, 52% of all MGUS cases were identified in 40‐ to 49‐year‐old donors (Figure 1). The median age at donor request creation of the MGUS cases was 43 years (age range: 21–60 years). In comparison, the median age of all requested donors in the observed period was 28 years.

**FIGURE 1:**
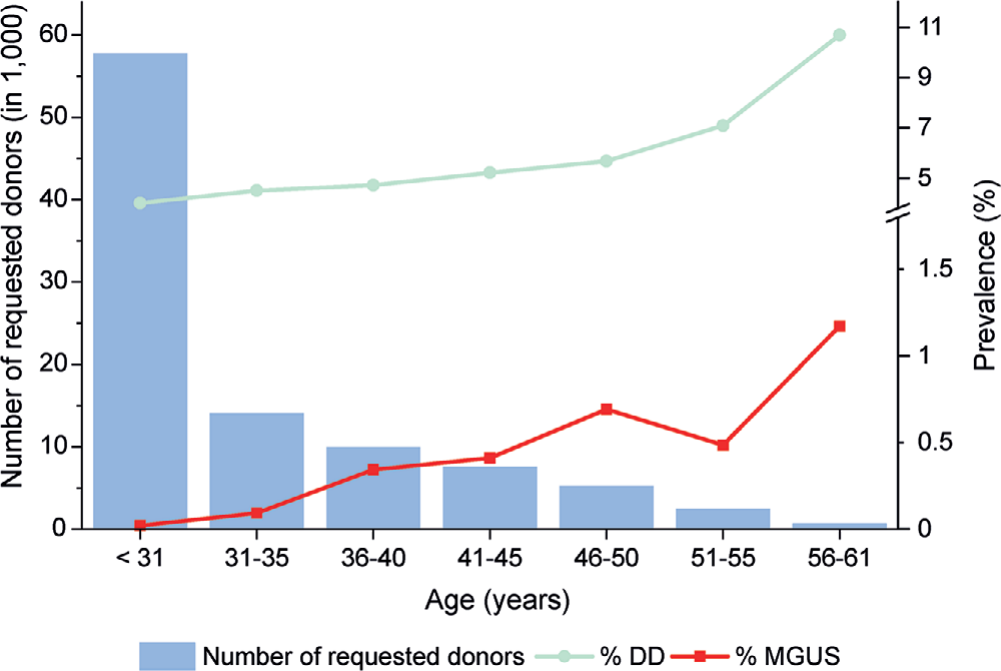
Prevalence of monoclonal gammopathy of undetermined significance (MGUS) and donor deferrals (DD) in percent, per number of requested donors in the respective age group.

Of the 143 donors who were not cleared due to an MGUS diagnosis, only four cases were detected with total serum protein above the reference range (≥ 84 g/L). Therefore, according to our data, testing of total protein alone was not suited to exclude monoclonal gammopathy in HSC donors since more than 97% of MGUS cases would have remained undetected if total serum protein concentration without SPEP had been used as a diagnostic measure. Monoclonal IgG kappa or lambda gammopathies were by far the most frequently diagnosed (76%, 109 cases), whereas IgM (11%, 16 cases) and IgA gammopathies (9%, 13 cases) were diagnosed less frequently. The rarest subclass was a biclonal gradient (IgG and IgA), which was detected only in two donors. In three cases no specific information was provided.

In the further course, normal SPEP/IFE results were documented in 17 donors (12%) several months after abnormal paraprotein detection during the physical examination. Forty‐five donors (31%) confirmed the MGUS diagnosis after consulting another doctor, whereas 81 donors (57%) did not disclose information about their MGUS status afterward.

In addition, electrophoresis revealed 23 cases of antibody deficiency (0.5% of donor non‐clearances). These cases would have remained undiagnosed otherwise and could have posed an additional risk for the HSC donor and recipient.

3

Overall, our data revealed 0.15% MGUS cases in our study population which is consistent with data from other population cohorts, ranging from 0.05% to 6.1% depending on age, ethnicity, and geographic area [10]. Individuals requested for HSC donation are generally a positively selected population group that is younger and healthier compared to the general population, which explains why the incidence of MGUS that we observed is at the lower end of the reported range.

According to our analysis, the vast majority of MGUS cases (139/143; 97.2%) would not have been detected without SPEP/IFE, and the affected donors would most likely have donated HSC. As a transmission risk of donor MGUS cannot be excluded, this is a strong argument in favor of routine testing for MGUS in the physical examination of potential HSC donors, especially as the costs and efforts of the corresponding tests (SPEP/IFE) are low.

A specific donation risk for clinically asymptomatic donors with MGUS remains unknown, thus the execution of SPEP/IFE does not have a direct positive effect on donor safety. Nevertheless, it is beneficial for the donor to be aware of the presence of MGUS to establish routine disease monitoring and prevent associated complications or consequential organ damage.

Taken together, the analysis of SPEP/IFE improves the quality and safety profile in HSC donor clearance during the physical examination and should become a standard diagnostic for potential HSC donors prior to donation wherever the medical infrastructure allows, specifically for older donors (> 40 years).

## AUTHOR CONTRIBUTIONS

Laura Kilinc and Thilo Mengling conceptualized and wrote the paper. Burkhardt Schleipen, Deborah Buk, Isabel Auer, and Thilo Mengling collected data. Laura Kilinc, Burkhardt Schleipen, Karen Ende, and Thilo Mengling analyzed data. Karen Ende and Alexander H. Schmidt contributed to the writing of the manuscript. All authors reviewed and approved the final manuscript.

## CONFLICT OF INTEREST STATEMENT

The authors declare no conflict of interest.

## FUNDING INFORMATION

The authors received no specific funding for this work.

## ETHICS STATEMENT

The authors have confirmed ethical approval statement is not needed for this submission.

## PATIENT CONSENT STATEMENT

The authors have confirmed patient consent statement is not needed for this submission.

## CLINICAL TRIAL REGISTRATION

The authors have confirmed clinical trial registration is not needed for this submission.

## Data Availability

The data collected for this study will be made available upon reasonable request; please contact the corresponding author Thilo Mengling: tmengling@dkms.de.
